# Thyroid disease and cancer in kidney transplantation: a single-center analysis

**DOI:** 10.1186/s12893-018-0408-1

**Published:** 2019-04-24

**Authors:** Massimiliano Veroux, Giuseppe Giuffrida, Salvatore Lo Bianco, Matteo Angelo Cannizzaro, Daniela Corona, Alessia Giaquinta, Chiara Palermo, Fausto Carbone, Anna Carbonaro, Maria Teresa Cannizzaro, Rossella Gioco, Pierfrancesco Veroux

**Affiliations:** 1 Vascular Surgery and Organ Transplant Unit, Unit of Endocrine Surgery, Department of Medical and Surgical Sciences and Advanced Technologies, University Hospital of Catania, Via Santa Sofia, 84 95123 Catania, Italy; 2 Manchester Royal Infirmary Organ Transplant Unit, Manchester, UK; 3 Unit of Endocrine Surgery, University Hospital of Catania, Catania, Italy; 4 Unit of Endocrine Surgery, Department of Medical and Surgical Sciences and Advanced Technologies, University Hospital of Catania, Catania, Italy; 5 Vascular Surgery and Organ Transplant Unit, Department of Medical and Surgical Sciences, University Hospital of Catania, Catania, Italy; 6 Vascular Surgery and Organ Transplant Unit, University Hospital of Catania, Catania, Italy; 7 Radiology Unit, University Hospital of Catania, Catania, Italy

**Keywords:** Thyroid, Goiter, Kidney transplantation, Cancer, Thyroid nodule, Papillary, Follicular, Fine needle aspiration citology

## Abstract

**Background:**

Thyroid diseases are frequent in patients with end-stage renal disease, but data on renal transplant recipients are conflicting. This study evaluated the incidence of thyroid disease and cancer in a population of kidney transplant recipients performed in a single center.

**Methods:**

Seven hundred sixty patients receiving a kidney transplantation between January 2000 and October 2017 were followed with thyroid ultrasonography to determine nodules together with thyroid hormone levels. Ultrasound-guided fine-needle aspiration citology (FNAc) was performed to the nodules > 10 mm .

**Results:**

Two hundred four patients (26.8%) patients demonstrated functional or morphologic changes in the thyroid gland compared with pre-transplant period. Among the 204 patients with newly diagnosed thyroid disease, 165 patients had single or multiple nodular lesions less than 1 cm in diameter, and were followed yearly. Nodule size progression was observed in 23 patients (13.9%), and they underwent a FNAc. A total of sixty-two patients (30.3%) underwent FNAc. The biopsy samples were cytologically interpreted as benign in 20 patients (32.2%), suspicious in 40 patients (64.5%), or at high risk of cancer in 2 patients (3.2%). Forty-two patients underwent total thyroidectomy. At histological examination, 18 patients had a thyroid cancer (papillary cancer in 17 patients, follicular cancer in one). Thyroid cancer was more frequent in male patients with a mean time from transplant to diagnosis of 5.6 years. At a mean follow-up was 8 ± 1.2 years, all patients are alive with a normal functioning graft.

**Conclusions:**

Thyroid diseases are common in transplant recipients. Thyroid disease may evolve after transplantation, probably as a consequence of immunosuppression. A complete evaluation of thyroid disease is mandatory in kidney transplant recipients because early diagnosis and appropriate treatment of thyroid disease and cancer may significantly decrease the morbidity and mortality in these patients.

## Background

Kidney transplantation is the preferred replacement therapy for patients with end-stage renal disease (ESRD). However, long-term immunosuppression may be responsible of a variety of complications, including the development of thyroid disease and cancer [1, 2]. Many studies have shown that, in kidney transplant recipients, the most common thyroid imbalance is a low T3 syndrome with FT3 levels generally within the normal limits [2–5], and this may have an impact on graft survival [5, 6]. Moreover, thyroid disease in kidney transplantation has been identified as a risk factor for cardiovascular disease (CVD) and a predictor of mortality [7].

Thyroid diseases may worsen after transplantation, mainly due to a consequence of the immunosuppression and this may probably explain the increased risk of thyroid cancer after transplantation [2, 8–12]. Interestingly, the risk of cancer in patients with ESRD is higher during dialysis period than during functioning kidney transplant intervals [13]. Taken together, these data suggest that a periodical evaluation of thyroid function should be recommended in kidney transplant recipients, but there are few studies investigating the rate of thyroid disease in kidney transplant recipients.

This study evaluated the incidence of thyroid disease and cancer in a population of kidney transplant recipients performed in a single center.

## Methods

This retrospective study included 760 patients who received kidney transplantation between January 2000 and October 2017. All ESRD patients, to be included in the waiting list, underwent ultrasonography of the neck and complete assessment of thyroid function according to serum levels of FT3, FT4, and TSH, as previously described [2]. In brief, all patients with a benign thyroid disease (nodular goiter or thyroiditis) were considered eligible for kidney transplantation after treatment of the disease, while patients with a diagnosis of thyroid cancer were considered eligible for kidney transplantation after at least 2 years of negative follow-up [2].

Kidney transplant recipients received a standard three-drug immunosuppressive therapy, with or without induction therapy with anti-interleukin-2 receptor antibodies (Simulect, Novartis, Basel, Switzerland) or with antithymocyte globulin (ATG-Fresenius, Fresenius, Bad Homburg, Germany), basing on both donor and recipient characteristics, as previously described [14].

After transplantation, in all transplant recipients Free T3 (normal range 1.8–4.2 pg/mL) and free T4 (normal range 0.8–1.9 ng/dL) levels were measured on a yearly basis and an ultrasonography of the neck was performed every 2 years. Anti-thyroid peroxidase antibody, anti-thyroglobulin antibody (Anti Tg), and serum TSH levels were also measured on a yearly basis. All patients with an isoechogenic solid thyroid nodule greater than 1 cm in diameter underwent fine-needle aspiration cytology (FNAc). Each cytological specimen was smeared on a slide immediately after aspiration. Thyroid nodules were classified according to the Italian cytological classification [15].

All patients with high levels of FT3 and FT4, low levels of TSH, and clinical signs or symptoms of hyperfunction of the thyroid gland (eg, tachycardia, flushing, or anxiety) underwent scintigraphy with iodine 131.

Surgical treatment was performed in kidney transplant recipients with a diagnosis of thyroid cancer or an uncertain diagnosis at FNAc, follicular adenoma, Plummer adenoma, or multinodular goiter and compressive symptoms on adjacent organs. Patients with benign disease, cystic lesions, or thyroid nodules smaller than 1 cm were followed up on yearly basis to evaluate thyroid function and perform ultrasonography [2].

### Statistical analysis

Results and patients characteristics are reported as mean ± SD. Comparison of means and percentages was estimated by the unpaired two Student’s t-test or Mann-Whitney U test, as appropriate. *p* value < 0.05 was considered as statistically significant. All calculations were performed using SPSS, version 12.0.

## Results

Two hundred four patients (26.8%) patients demonstrated functional or morphologic changes in the thyroid gland compared with pre-transplant period. The demographical and clinical characteristics of patients are reported in Table 1. Clinical characteristics were similar among recipients developing a thyroid nodule compared to recipients who did not. Patients with thyroid nodule were more frequently younger with a longer time on dialysis and on waiting list for kidney transplantation, suggesting the role of time on dialysis on the development of thyroid disease. Type of immunosuppression did not have a role in the development of thyroid disease.

**Table 1:** Clinical characteristics of the study cohort

	With nodule (n=204)	Without nodule (n=556)	*P*
Male (%)	48%	47%	Ns
Living/Deceased	40/164	60/494	Ns
Age (years)	48 ±12.3	54 ±10.5	< 0.04
Cause of ESRD (n,%)			
Glomerulonephritis	95 (46.6)	265 (47.6)	Ns
Polycystic Kidney Disease	40 (19.7)	87 (15.7)	Ns
Diabetes	30 (14.7)	60 (10.8)	Ns
Other causes	39 (19.1)	144 (25.9)	Ns
Waiting list (months)	21 ±10.4	18.2 ±16.4	Ns
Pretransplant dialysis	32 ±16.3	29 ±22.3	< 0.05
Donor age	55.6 ± 15.1	54.2 ±18.1	Ns
Immunosuppression (n, %)			
Induction	60 (29.4)	160 (28.2)	Ns
TAC+MPA+Ster	170 (83.3)	470 (83)	Ns
CYA+MPA+Ster	20 (9.8)	60 (10.6)	Ns
Eve+MPA+Ster	24 (11.7)	36 (6.3)	Ns
BMI (KG/m^2^)	25 ±4.7	26.1 ±3.9	Ns
Serum Creatinine (mg/dL)	1.53 ±0.7	1.49 ±0.6	Ns

*ESRD* end-stage renal disease, *TAC* tacrolimus, *MPA* mycophenolic acid, *Ster* steroids, *CyA* cyclosporine, *EVE*, everolimus, *BMI* body mass index *NS*, not significant

Among the 204 patients with newly diagnosed thyroid disease, 165 patients had single or multiple nodular lesions less than 1 cm in diameter, and were followed yearly. Nodule size progression was observed in 23 patients (13.9%), and they underwent a FNAc.

A total of sixty-two patients (30.3%) underwent FNAc: Of the patients who had nodules, 58 patients had normal thyroid function, while clinical hyperthyroidism was diagnosed in 4 patients. The biopsy samples were cytologically interpreted as benign (TIR 2) in 20 (32.2%), suspicious (TIR 3A or B) in 40 patients (64.5%), or at high risk of cancer (TIR 4) in 2 patients (3.2%).

Two patients with clinical signs of hyperthyroidism at 131I scintigraphy had a hyperfunctional goiter and underwent left lobectomy.

Forty-two patients underwent total thyroidectomy. At histological examination, 18 patients had a thyroid cancer (papillary cancer in 17 patients, follicular cancer in one). Patients with thyroid cancer were more frequently male (15/18), with a median age of 42 ± 15.4 years. Mean time from transplant to diagnosis was 5.6 y (range 3–12, years). Multifocal cancer was observed in three patients, while microcarcinoma (< 1 cm) was observed in three patients. Four patients presented with lymph node metastasis at the time of diagnosis.

Adjuvant radioiodide treatment was applied in all patients with persistent high level of thyroglobulin after thyroidectomy. In patients with diagnosis of cancer, immunosuppressive therapy was slightly reduced without the need for a switching to another drug.

Three patients, with lymph node metastasis at time of diagnosis, had a local recurrence at 6, 10 and 11 months after thyroidectomy and underwent lymph node dissection. There were no signs of local recurrence or distant metastasis in the remaining 15 patients. At a mean follow-up was 8 ± 1.2 years, all patients are alive with a normal functioning graft.

## Discussion

Thyroid disease is frequent in patients with end-stage renal disease, but data on renal transplant recipients are conflicting. The uremia and the long-term immunosuppression may have a role in the increased incidence of thyroid carcinoma in renal transplant patients compared to general population [16].

However, very few studies investigated the incidence of thyroid disease in kidney transplant population. In a study by Łebkowska U et al., [17], the incidence of thyroid disease was evaluated by ultrasound-guided FNAb in 44 kidney transplant recipients. They found that all patients presented with a various degree of goiter, with a thyroid nodule in 43% of patients and a thyroid cancer in 11.4% of patients. Moreover, they found a significant association between cyclosporine level and the occurrence of goiter, confirming that higher level of immunosuppression may increase the risk of developing a thyroid cancer [17]. More recently, Gungor et al [16], reported a higher incidence of thyroid nodule (40%) and goiter (91.8%) in 122 kidney transplant recipients compared with the general population.

This study evaluated the incidence of thyroid disease among a population of 760 kidney transplantations performed in a single center. A newly diagnosed thyroid disease was reported in 240 patients (16.8%). Most of patients had thyroid nodule < 1 cm in diameter (80.8%), but 18 patients developed a thyroid cancer. Younger patients, with long-term dialysis and waiting list are at higher risk for developing a thyroid cancer, but there was no significant association with the type of immunosuppression adopted, suggesting that the cumulative dose of immunosuppression rather than the type of immunosuppression may have a significant role in the increased risk of thyroid cancer in kidney transplant patients.

Organ transplant recipients have higher risk of developing a post-transplant cancer. However, this risk is particularly high for virus-linked neoplasm, in which some oncogenic viruses such as human herpesvirus-8 or Epstein-Barr virus are activated by immunosuppression causing the post-transplant lymphoproliferative disorders. In contrast, cancers that are more frequently observed in the general population (colon, breast, lung, etc) are not related to oncovirus, and their incidence is not significantly increased after renal transplantation [2, 8–13].

There are few data in literature on the incidence of thyroid cancer in kidney transplant recipients. A study from Australia and New Zealand [18] evaluated the incidence of thyroid cancer among a cohort of 10,689 renal transplant recipients, and found that 23 patients (0.22%) were diagnosed with a post-transplant thyroid cancer. The median age in the renal transplant group with thyroid cancer was 48.2 years. Interestingly, thyroid cancer was more common in male sex, as confirmed in our study, and 43% of patients presented lymphatic metastases at the time of the primary diagnosis. More recently, Karamchandani et al [8] showed that the risk of thyroid cancer is significantly higher following kidney transplantation (SIR 6.94), and most cancers are of papillary type and appear about 6 years following transplant. More recent studies reported a SIR of 1.9 [10], while Kitahara et al [11] reported an incidence rate ratio of thyroid cancer among kidney transplant recipients of 1.26, being more frequent when the cause of ESRD was hypertensive nephrosclerosis and in patients with longer prior dialysis before transplantation. Overall, thyroid cancer was associated with a modest increase of death (hazard ratio 1.33).

The incidence of thyroid cancer in our series was 2.3%. All patients but one had a papillary cancer. Although rarely observed, follicular neoplasm may occur even in kidney transplant recipients, and pre-operative diagnosis is often difficult due to the limited sensitivity of FNAc, mandating a surgical treatment in most patients [19]. A lymphatic metastasis was observed in four patients, and cancer occurred more frequently in men at a mean time of 5.6 years after transplantation. In general population, lymph nodal involvement is very common in differentiated thyroid cancer, with micrometastases observed in up to 80% of papillary thyroid cancers [20], but this higher incidence was not observed in our series, probably due to early diagnosis. Thyroid cancer recurred in three patients, but patient and graft survival were not significantly affected and all patients are alive at last follow-up. Again, the chronic immunosuppression may be likely related to the risk of thyroid cancer, as demonstrated by the occurrence of cancer many years after the transplantation. However, recent studies have demonstrated that thyroid cancer can occur even early in the post-transplantation follow-up and its histologic behavior is often more aggressive than in the general population [2].

Although the more aggressive behavior of thyroid cancer in kidney transplant recipients, there was no cancer-related mortality in our experience. We could suggest, therefore, a yearly-based neck sonography in all kidney transplant recipients together with the evaluation of thyroid functionality. In all patients with a nodule increasing in size or suggestive for thyroid cancer at citology, a total thyroidectomy should be performed.

The main limit of this study is that, although performed in a large cohort of patients, many patients could be lost at follow up, due to graft loss or patient death, so that the incidence of thyroid disease and cancer could be underestimated.

## Conclusions

Thyroid diseases are common in transplant recipients. Thyroid disease may evolve after transplantation, probably as a consequence of immunosuppression. A complete evaluation of thyroid disease in kidney transplant recipients is mandatory because early diagnosis and appropriate treatment of thyroid disease and cancer may significantly decrease the morbidity and mortality in these patients.

## Abbreviations

ATG: Antithymocyte globulin
CVD: Cardiovascular disease
ESRD: End-stage renal disease
FNAb: Fine-needle aspiration biopsy
FNAc: Fine-needle aspiration cytology
TSH: Thyroid stimulant hormone
